# The effect of age-related sensorimotor changes on step-down strategy: a predictive simulation study

**DOI:** 10.1038/s41598-025-14422-0

**Published:** 2025-08-21

**Authors:** Lucas Schreff, Niels F. J. Waterval, Marjolein M. van der Krogt, Daniel F. B. Häufle, Roy Müller

**Affiliations:** 1 Department of Orthopedic Surgery, Klinikum Bayreuth GmbH, Bayreuth, Germany; 2 Bayreuth Center of Sport Science, University of Bayreuth, Bayreuth, Germany; 3 Hertie Institute for Clinical Brain Research and Center for Integrative Neuroscience, Tübingen, Germany; 4 Amsterdam Movement Sciences, Rehabilitation and Development, Amsterdam, The Netherlands; 5 Rehabilitation Medicine, Amsterdam Movement Sciences, Amsterdam UMC, University of Amsterdam, Amsterdam, The Netherlands; 6 Department of Rehabilitation Medicine, Amsterdam UMC, Vrije Universiteit Amsterdam, Amsterdam, The Netherlands; 7 Institute for Computer Engineering, Heidelberg University, Heidelberg, Germany; 8 Center for Bionic Intelligence (BITS), Tübingen Stuttgart, Germany; 9 Universitätsklinikum Erlangen, Friedrich-Alexander-Universität Erlangen-Nürnberg, Erlangen, Germany

**Keywords:** Human gait, Neuromusculoskeletal model, Anticipation, Optimization, Aging, Elderly walking, Biomedical engineering, Motor control, Computational models, Neural ageing, Ageing

## Abstract

Humans adjust neuromuscular control in anticipation of a step-down during walking. Due to age-related sensorimotor changes, older adults may require adaptation of this control to step-down safely. We used predictive simulations to investigate how muscle weakness and delayed neural transmission affect anticipatory control during step-down. Five model variants were developed: a default model, two with muscle strength reduced to 80% and 60%, and two with neural delays increased by 20% and 40%. For each model, we tested two strategies in the trailing leg during the last contact before step-down: reduced soleus activity (SOL strategy) and increased hamstring activity (HAM strategy). We systematically varied step-down height and anticipatory control levels. For the SOL strategy, both muscle weakness and neural delay reduced the maximum feasible step-down height, with muscle weakness requiring more precise adjustments. The HAM strategy was mainly affected by neural delay and showed less sensitivity to control precision. While the SOL strategy generally performed better, the HAM strategy was more robust under severe weakness. These results suggest that the HAM strategy may benefit individuals with progressive sensorimotor decline, while maintaining SOL strategy applicability—e.g., through strength training—could help maintain its benefits. Further investigations are needed to confirm this.

## Introduction

With increasing age, the risk of falling while walking rises (e.g^1–3^). Chance of falling is highest when obstacles are involved, such as uneven grounds^4^ that require gait adaptation. The increased risk of falling is attributed to various age-related sensorimotor changes, such as muscle weakness due to loss of muscle mass^5,6^ and delayed neural signal transmission^7^. To better understand the causal relationships between these changes and the risk of falls, an isolated examination of age-related factors is necessary^8,9^. While considering isolated sensorimotor changes is challenging in experimental studies (e.g^10^), computational models excel at predicting their impact on motion control (e.g^11–13^).

**Fig. 1:**
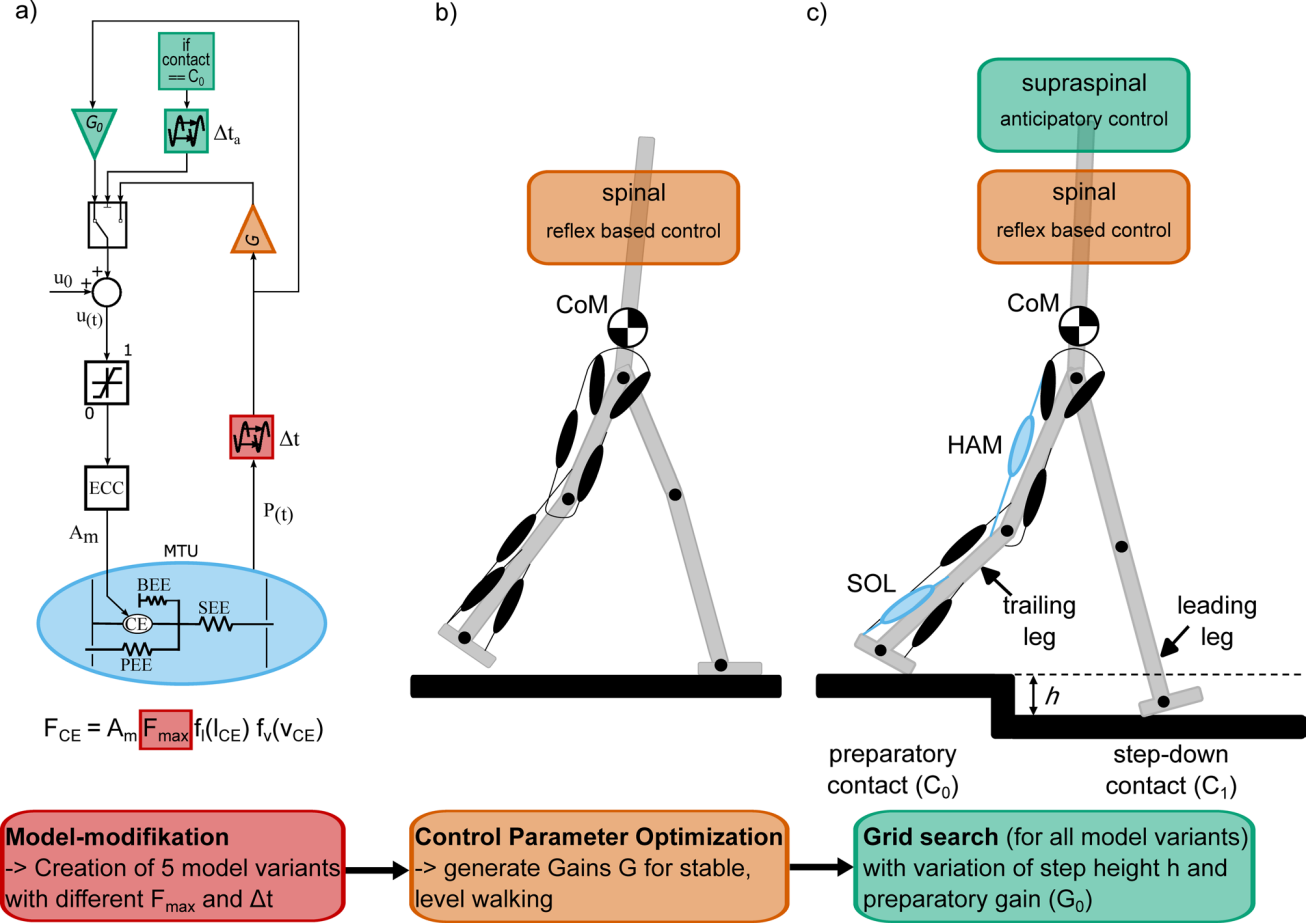
Overview of the methodological approach for this study. (**a**) Five model variants were created with different maximum isometric muscle forces (F_max_) and neuronal signal delays (Δt). (**b**) Their control parameters were optimized to achieve steady-state level walking, (**c**) and the step-down robustness of two anticipatory strategies was evaluated across each model variant. The model variants are based on the established neuromuscular reflex model of Geyer and Herr^14^ which consists of seven segments connected by hinge joints. Each leg contains seven Hill-type muscle-tendon units. The gluteus (GLU), hip flexors (HFL), hamstrings (HAM), vasti (VAS), gastrocnemius (GAS), soleus (SOL), and tibialis anterior (TA) are exemplarily shown in the trailing leg. Muscle stimulation patterns are generated by reflex-based signals, primarily via proprioceptive muscle force and length feedback. The corresponding reflex loop is depicted in the block diagram (**a**). The muscle-tendon unit (MTU) provides the feedback signal P(t). This feedback signal is time-delayed with Δt, gained by the feedback gain G and added to a constant stimulation bias (u_0_). The resulting stimulation signal (u_t_) is limited to values between 0 and 1. Afterwards, excitation–contraction coupling (ECC) time constants are used to determine the muscle activity (A_m_). The force generated by the contractile element (F_CE_) depends on muscle activity, maximum isometric muscle force, the force-length relationship of the CE (f_l_(l_CE_)), and the force-velocity relationship of the CE (f_v_(v_CE_)). In addition to the spinal reflex-based control (highlighted in orange), the block diagram (**a**) depicts the supraspinal anticipatory control (highlighted in green), applied exclusively during preparatory contact in the trailing leg (**c**). To apply the anticipatory SOL and HAM strategies, the reflex gain G was switched to the preparatory gain G_0_. Both strategies were started when the corresponding muscles exceed 10% activity during the preparatory contact. Therefore, the time delay of the preparatory gain adjustment (Δta) after heel strike was 200 ms for the SOL strategy and 100 ms for the HAM strategy.

One model that can predict the kinematics and dynamics of human gait is the walking model by Geyer and Herr^14^ which relies on proprioceptive feedback loops for neuromuscular control ( ≙ reflex based control^15^. Extensions of this model have been used to analyze the effects of age-related sensory-motor changes on metabolic cost of walking^16^ and sensorimotor strategies to increase robustness in step-down perturbations^17,18^. In these extensions, robustness was improved either by adding a preactivation reflex^17^ or by incorporating an additional feedforward control mechanism^18^. However, the impact of age-related changes on suitable anticipatory control strategies during stepping down (see Fig. 1) has not been investigated.

Recently, we showed that anticipatory strategies are effective in increasing the maximum step-down height of the model^19^. We tested various anticipatory strategies by selectively increasing or decreasing the reflex gains of several muscles exclusively during the last stance phase before the step-down ( ≙ preparatory contact phase). Specifically, we modulated the gains of the hamstrings (HAM), vasti (VAS), gastrocnemius (GAS), soleus (SOL), and tibialis anterior (TA). Of all tested strategies, two adaptations most clearly increased the robustness of the model. We referred to these as the SOL and HAM strategies. In the SOL strategy, the feedback loop of the SOL in the trailing leg (Fig. 1) was modified by reducing the SOL reflex gain during the preparatory contact phase. Due to the reflex control of the model, this affects all muscle activities but mainly reduces SOL activity in the trailing leg. As a result of these muscular adaptations, the ankle and knee joint of the trailing leg were (dorsi-) flexed more during the preparatory contact phase, lowering the center of mass (CoM) level ( ≙ vertical distance between CoM and floor) at heel strike of the leading leg in the step-down contact phase. In the HAM strategy, the feedback loop of the HAM in the trailing leg was modified by increasing the HAM reflex gain anticipatorily during the preparatory contact phase. This mainly resulted in an increased HAM activity, a more extended trailing leg and consequently in a higher CoM level. Experimental data demonstrates that young adults use the SOL strategy^20,21^ while older adults seem to switch to use a strategy similar to the HAM-strategy. Older adults show higher co-contractions surrounding the knee and ankle joints, resulting in a more extended trailing leg during the stair descent^22,23^. The use of the HAM strategy by older people is also supported by the observation that they use the stronger knee flexors and extensors more than the plantar flexors when they descending stairs^24,25^.

Currently, it is being discussed why older adults seem to change their step-down strategy. As a step-down is a difficult task with a high risk of falling, understanding the underlying reasons for these alterations is a first step to develop interventions, such as training programs or assistive devices. We expect that the change in step-down strategy is partly the result of decline in muscle force and neural delay. Co-contraction, which results from using the HAM strategy (a higher and longer hamstring activity) but not in the SOL strategy, likely improves joint stability^26,27^ - a mechanism known to enhance stability during walking on slippery ground and uneven terrain^28^. Hence, we expect that greater muscle weakness may contribute to a shift in strategy. In addition, neural delays could also be one of the reasons why older adults may prefer the HAM strategy: increased co-contraction in the trailing leg allows for a more controlled lowering of the center of mass^22^ reducing its downward acceleration and, consequently, the need for rapid reflex-based corrections in the leading leg.

With experiments it is extremely difficult to determine whether muscle weakness, or neural delay contributes to the change in step-down strategy in older adults, since physiological parameters decline simultaneously. Furthermore, determining the point of change is very difficult as muscle weakness or neural delay cannot be systematically altered. To test our hypothesis that both muscle weakness and neural delay could potentially explain the change in step-down strategy we applied our predictive simulations.

The purpose of this study is to investigate the effects of age-related muscle weakness and delayed neural signal transmission on the performance of the anticipatory SOL and HAM strategies when performing step-downs using predictive simulations. We expected that a reduced muscle force (MF) or neural delay (ND) would decrease the robustness against falls when stepping down compared to a model without age-related sensorimotor changes. However, the HAM strategy is expected to be less affected by such changes and may therefore offer greater robustness in individuals with sensorimotor impairments.

## Methods

In a nutshell, we performed predictive simulations that were built upon the established reflex-based walking model by Geyer and Herr^14^. From this model, we created five models reflecting the neuromuscular changes found in older adults by reducing maximal muscles forces and increasing neuronal delays (Fig. 1a). We optimized the neuronal control parameters for each model variant to generate steady-state level walking (Fig. 1b), and then extended these model variants with an anticipatory controller (Fig. 1c and green extension of Fig. 1a) to deal with step-down perturbations^19^. Next, we performed a grid search by systematically varying step-down height and anticipatory control parameters. This allowed us to evaluate and compare the step-down robustness of two different anticipatory control strategies across the different “aged” model variants.

### Control parameter optimization for different model variants

Our study builds on the neuromusculoskeletal model developed by Geyer and Herr^14^. The reflex model employs a feedback control mechanism ( ≙ reflex based control, Fig. 1a marked in orange) that relies mainly on proprioceptive muscle length and muscle force reflex loops to stimulate the seven muscles of each leg (gluteus (GLU), hip flexors (HFL), hamstrings (HAM), vasti (VAS), gastrocnemius (GAS), soleus (SOL), and tibialis anterior (TA)). To investigate walking with muscle weakness (reduced maximal isometric force F_max_) and delayed neural signal transmission (Δt), we created five model variants: A default model with the original muscle force and delay parameters proposed by Geyer and Herr^14^, which are based on experimental data of real individuals^29^; This model can be considered a healthy young subject^16^. Two model variants with maximal isometric muscle forces reduced to 80% of F_max_ (80_MF-model) and to 60% (60_MF-model) of all muscles equally (reduction by 20%, and 40% respectively); and two model variants where the neural signal transmission of all nerve pathways was extended by 20% (120_ND-model) and 40% (140_ND-model), respectively. An overview of the muscle forces and neural signal transmission delays for each model variant is provided in Table 1.

**Table 1:** Overview of the maximum isometric muscle forces (F_max_) and neural signal transmission delays Δt for all model variants.

Model variants	GLU	HFL	HAM	VAS	GAS	SOL	TA
Default							
F_max_ [N]	1500	2000	3000	6000	1500	4000	800
Δt [ms]	5.0	5.0	5.0	10.0	20.0	20.0	20.0
80_MF							
F_max_ [N]	1200	1600	2400	4800	1200	3200	640
Δt [ms]	5.0	5.0	5.0	10.0	20.0	20.0	20.0
60_MF							
F_max_ [N]	900	1200	1800	3600	900	2400	480
Δt [ms]	5.0	5.0	5.0	10.0	20.0	20.0	20.0
120_ND							
F_max_ [N]	1500	2000	3000	6000	1500	4000	800
Δt [ms]	6.0	6.0	6.0	12.0	24.0	24.0	24.0
140_ND							
F_max_ [N]	1500	2000	3000	6000	1500	4000	800
Δt [ms]	7.0	7.0	7.0	14.0	28.0	28.0	28.0

The extent of reduced muscle force and delayed neural signal transmission is similar to the study of Song and Geyer^16^ and based on experimentally data^5–7,30–32^. Age-related muscle loss of approximately 3% per year after the age of 60 has been reported^30^ resulting in an overall strength reduction of ~ 17% to ~ 41% when comparing individuals younger than 40 years with older individuals (> 40 years)^32^. Similarly, aging is associated with reduced nerve conduction velocity, including in reflex pathways such as the H-reflex. Median sensory nerve conduction velocity declines by more than 15% when comparing individuals aged 15–30 to those aged 46–60 ^31^. The 20% increase in neural delay applied in our simulations is thus within the range of experimentally observed age-related declines. The 40% increase, while somewhat higher, was included to explore more pronounced impairments and to enable a consistent comparison with the corresponding levels of muscle weakness. Using the same scaling factors (20% and 40%) for both impairments allows us to systematically evaluate their relative contributions to strategy selection.

For each model variant, we optimized 12 control parameters ( ≙ reflex gains G, Fig. 1a marked in orange) during level walking (Fig. 1b) with the covariance matrix adaptation evolution strategy (CMA-ES)^33^. As initial parameters for the optimization, we always used the original control parameters of Geyer and Herr^14^. The cost function contained two stages:

1 $$J = \left\{ {\begin{array}{*{20}l} {50 - x_{{fall}} } & {{\text{a)~if~fall}}~} \\ {c_{1} (\frac{1}{T}\mathop \sum \limits_{m} \smallint A_{m} ^{2} {\text{d}}t) + c_{2} \left| {\Delta v} \right| + c_{3} \left( {\Delta l} \right) + c_{4} \left( {tc_{{diff}} } \right) + c_{5} \left( {S_{{diff}} } \right)} & {{\text{b}}){\text{~if~not}}} \\ \end{array} } \right.$$

In the first stage (see 1a), we aimed to find a stable level gait pattern. X_fall_ represents the distance traveled by the model without falling. The value of 50 in 1a was chosen to ensure that the calculated costs of the first stage were always higher than those of the second stage (1b).

After achieving a level gait pattern without falling for 20 s, Eq. 1b was responsible to minimize five different target variables. These variables were weighted using factors c1 to c5 (c1 = 0.17, c2 = 0.8, c3 = 0.8, c4 = 0.05, c5 = 0.15). The first objective was to minimize muscle activity (integration of squared muscle activity $\:(\frac{1}{T}\sum\:_{m}\int\:{{A}_{m}}^{2}\text{d}t)\:$over the time span T of six consecutive steps), which is important to generate a humanlike gait pattern (first term of Eq. 1b). This was in line with Song and Geyer^16^ who also used the integration of squared muscle activation to optimize gaits with age-related sensorimotor changes. All other target variables of Eq. 1b were constraints to optimize towards a steady state gait with a speed *v* of 1.2 m/s, step length *l* of 0.75 m, and toe clearance *tc*_*diff*_ between 1.2 and 1.6 cm. These constraints were added to make the resulting gaits as comparable as possible amongst each other, but also to elderly gait (e.g^34^), with slower walking and shorter steps than the original model (v_sim_=1.38 m/s; l_sim_=0.78 m) by Geyer and Herr^14^. We also added the minimum toe clearance (tc), defined as the local minimum distance between the foot and ground during the swing phase of gait (e.g^35^) which is an important factor for trip-induced falls^36^ and should be comparable between the model variants.

The last term in Eq. 1b ascertains steady state: as in Schreff et al. (2023), a gait was considered steady if the maximum pairwise difference (ΔS) between the margin of stability^37,38^ at six consecutive heel-strikes was less than 0.75 cm. Equation 2 indicates that only ΔS higher than 0.75 cm generates costs during optimization.

2 $$S_{{diff}} = \left\{ {\begin{array}{*{20}l} {\Delta S - 0.75} & {{\text{if}}~\;\Delta S > 0.75} \\ 0 & {{\text{if}}\;~\Delta S < 0.75} \\ \end{array} } \right.$$

The optimization with the CMA-ES was performed for 1500 generations in each optimization. During the optimization runs, typically all terms in the cost function reached (nearly) zero, except the muscle activity term. The model variants were implemented in Matlab^®^ Simulink^®^ R2021a and the simulations were performed with the ode15s solver (max. step size of 10 ms, relative and absolute error tolerance of 10^− 3^ and 10^− 4^, respectively).

### Extended model for anticipatory control

In our previous study^19^ we extended the reflex based control (Fig. 1 marked in orange) of the original model to include an anticipatory control strategy (Fig. 1 marked in green). This anticipatory control switches the feedback gain (G) to the preparatory gain (G_0_, Fig. 1a) for individual muscles, thereby increasing or decreasing the proprioceptive feedback signals. The anticipatory strategy modifies the muscle stimulation exclusively during the preparatory contact phase (C_0_, Fig. 1c) in the trailing leg.

For the step-down simulations of this study, we used this control extension to implement two anticipatory strategies in all of our optimized model variants: The SOL strategy involved reducing the preparatory gain of the soleus muscle (G_0_SOL_), which decreased its muscle activity in preparation for the step-down. Conversely, in the HAM strategy, an increase in the preparatory gain of the hamstrings (G_0_Ham_) led to heightened muscle activities. Both strategies were found to increase step-down robustness in a healthy model in our previous study^19^.

### Model analysis

In the simulations, the model variants (default model, models with muscle weakness 80_MF, 60_MF and delayed neural transmission models 120_ND, 140_ND) walked for ten seconds before reaching the step-down. A trial was considered successful if the model returned to a stable walking pattern after step-down, determined by the fact that the model could take five steps after step-down, and the last step length exceeded 0.65 m, to rule out cases in which the fifth step corresponded to tripping. We performed a grid search to identify successful step-down walking patterns for each model variant. For this purpose, we systematically varied step-down height (h), between 0 and 20 cm in steps of 1 cm, and preparatory gain. For each height, we adjusted the preparatory gain of the trailing leg according to the selected strategy (SOL or HAM).

For the SOL strategy, we reduced the preparatory gain compared to the reference normal walking gain. The investigated gain value range was G_0_SOL_= [x/F_maxSOL_; G_SOL_]. The lower bound x was scaled with respect to the model-specific maximum isometric force of the soleus (i.e., 0.5 for models with 100% muscle force, 0.62 for 80_MF, and 0.74 for 60_MF) to ensure a consistent step size of 1.25 × 10^− 5^ across all model variants. For the HAM strategy, the gain was increased compared to the reference gain of normal walking for the specific model until a gain of 5.0 was achieved. The step size for the gain increase was 0.5. The range of tested gain adjustments was based on our findings in Schreff et al.^19^.

Additionally, to understand the consequences of muscle weakness and neural delay in the interaction with the anticipatory strategies in the different model variants, we analyzed the muscle activities as well as the knee and ankle kinematics of the trailing leg during the preparatory contact phase (C_0_) for the maximal step-down that was achieved by the model variants (12 cm for reducing muscle force, and 7 cm for increasing neural delay). As we qualitatively compare the maximum successful step-down height, the range of reflex gains leading to success, muscle activations and joint kinematics between different model variants, no statistical analyses were performed.

## Results

### Robustness of the model variants

The optimization runs produced stable, steady gait patterns for all five model variants. For the default model, the step-down height increased from 3 cm without anticipation to 19 cm when using the SOL strategy and 12 cm when using the HAM strategy (Table 2). All model variants (80_MF, 60_MF, 120_ND, 140_ND) benefit from SOL and HAM strategy (Table 2).

**Table 2:** Maximum rejected step-down heights h_max_ with and without anticipation for all model variants.

Model variants	h_max_ without anticipation [cm]	h_max_ with anticipation [cm]	
		SOL-Strategy	HAM-Strategy
default	3	19	12
80_MF	5	16	12
60_MF	4	14	16
120_ND	3	14	9
140_ND	3	13	7

**Fig. 2:**
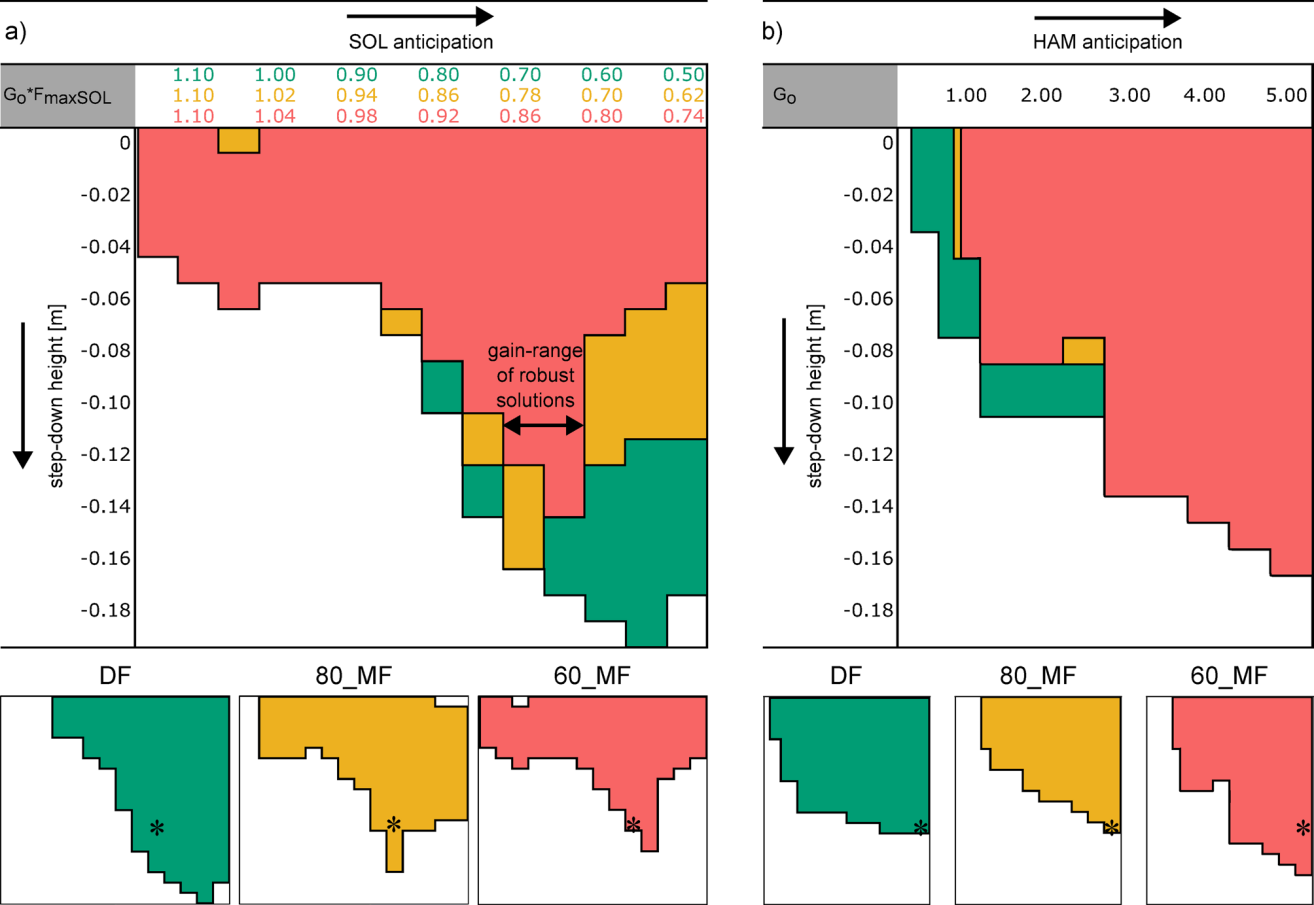
Effect of SOL and HAM anticipation strategies on walking robustness for different levels of muscle weakness. Colored areas indicate successful trials for various gain (G_0_) adaptions (x-direction) and different step-down heights h (y-direction). The results of the default model (green) and the models with reduced muscle force 80_MF (orange) and 60_MF (red) are depicted. In the SOL strategy (**a**), G_0_ is gradually reduced from the reference feedback gain (G) to analogously reduce muscle activity during the preparatory contact. In the HAM strategy (**b**), G_0_ is gradually increased from the reference feedback gain (G) to analogously increase muscle activity during the preparatory contact. The gain-range of robust solutions represents the number of gain adjustments that lead to successful trials for a certain step-down height. Trials marked with * were used for the analysis of muscle activities and joint kinematics in Fig. 4. Note: When specifying the gains for the soleus muscle, its maximal isometric force (F_maxSOL_) is included as a scaling factor. To ensure a consistent step size of 1.25 × 10^− 5^ for anticipatory gain adjustments across the three model variants (DF, 80_MF, and 60_MF), the x-axis values differ between model variants.

Regarding the SOL strategy, reducing the maximal muscle force led to a decrease in h_max_ from 19 cm (default) to 16 cm (80_MF) and 14 cm (60_MF) respectively (Table 2). In addition, the range of gain adjustments that led to successful trials for a certain step-down height ( ≙ gain-range of robust solutions, Fig. 2a), decreased with increasing muscle weakness (more narrow vertical area for larger step-down heights in the orange and red shaded area of Fig. 2a. For example, for a step-down of 12 centimeter, the default model could have a SOL gain between 0.75/F_maxSOL_ and 0.50/F_maxSOL_ (i.e., gain-range of robust solutions = 6,25 × 10^− 5^), while the 60_MF model could only successfully take such a step-down with SOL reflex gains of 0.86/F_maxSOL_ and 0.83/F_maxSOL_(i.e., gain-range of robust solutions = 1,25 × 10^− 5^).

In contrast to the SOL strategy, muscle weakness did not decrease h_max_ when applying the HAM strategy. Compared with the default model (h_max_ = 12 cm with HAM strategy), the 80_MF model variant had a similar performance (h_max_ = 12 cm for both models), while the 60_MF-model even had a 4 cm higher maximal step-down (h_max_ = 16 cm). Additionally, the gain-range of robust solutions did not decrease with the HAM strategy (Fig. 2b), and maximum step-down heights could be rejected for all model variants with maximum hamstring gains (e.g., full hamstring activation, Fig. 4).

**Fig. 3:**
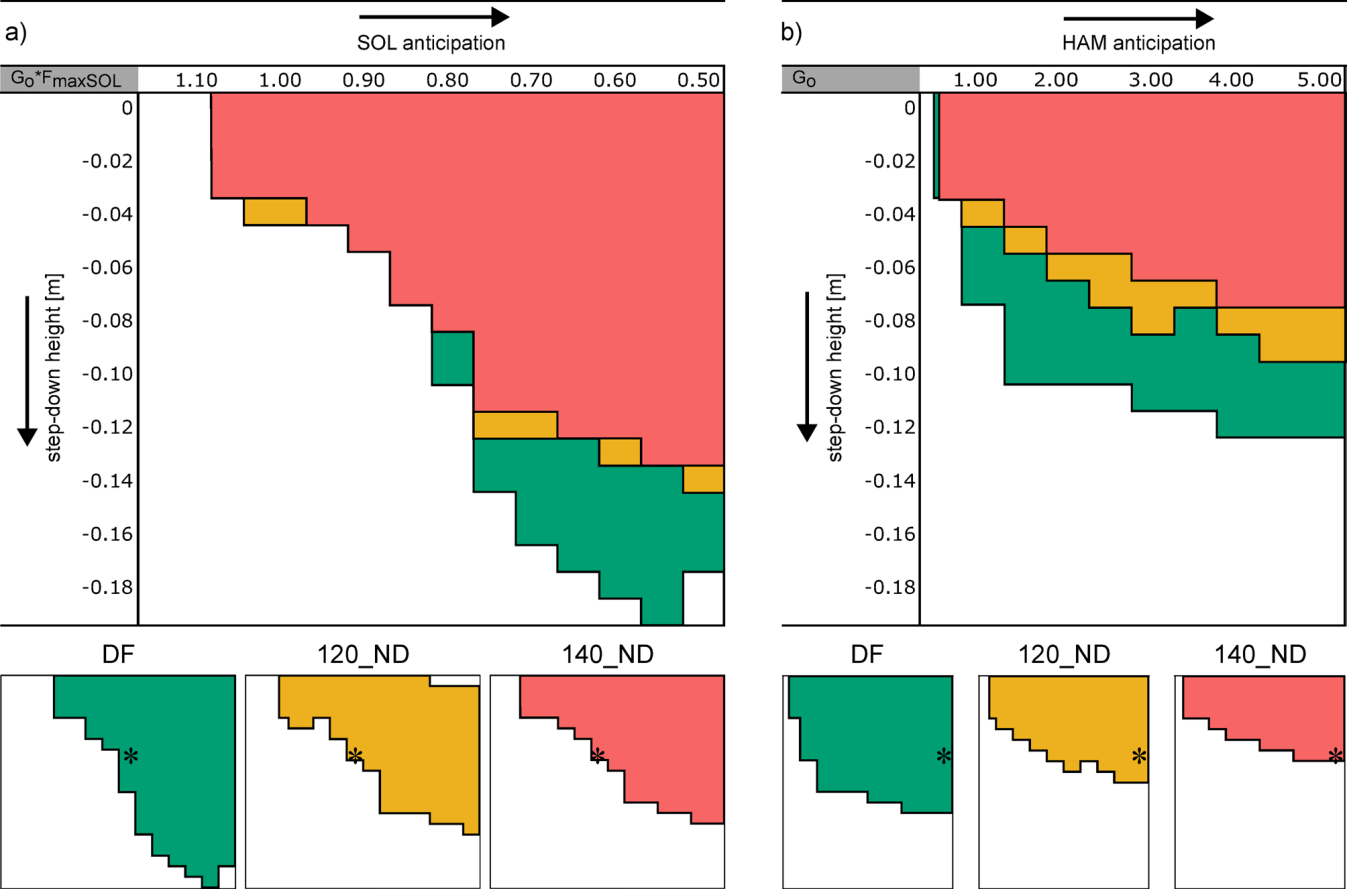
Effect of SOL and HAM anticipation strategies on walking robustness for different levels of neural delay. Colored areas indicate successful trials for various gain (G_0_) adaptions (x-direction) and different step-down heights h (y-direction). The results of the default model (green) and the models with delayed neural signal transmission 120_ND (orange) and 140_ND (red) are depicted. In the SOL strategy (**a**), G_0_ is gradually reduced from the reference feedback gain (G) to analogously reduce muscle activity during the preparatory contact. In the HAM strategy (**b**), G_0_ is gradually increased from the reference feedback gain (G) to analogously increase muscle activity during the preparatory contact. Trials marked with * were used for the analysis of muscle activities and joint kinematics in Fig. 5.

In the model variants with delayed neural signal transmission, the SOL strategy (Fig. 3a) resulted in a higher maximal step-down compared to the HAM strategy (Fig. 3b). Compared to the default model using the SOL strategy, increasing neural delay decreased h_max_ from 19 cm (default model) to 14 cm (120_ND-model) and 13 cm (140_ND-model) respectively. When applying the HAM strategy, h_max_ decreased from 12 cm (default model) to 9 cm (120_ND-model) and 7 cm (140_ND-model) respectively. The gain-range of robust solutions was not notably affected for both the SOL and HAM strategy.

### Anticipatory muscular and kinematic adaptations

**Fig. 4:**
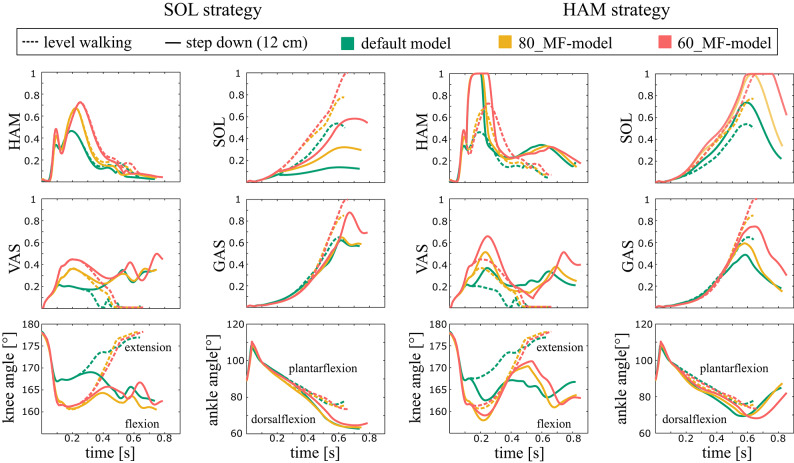
Muscle activities and kinematics during the preparatory contact phase (C_0_; 0 s ≙ heel strike) for the default and muscle weakness model variants. Different colors indicate the different muscle weakness levels. Solid vs. dashed lines compare 12 cm step-down vs. level walking, respectively. Here, we show only the results for the 12 cm step-down, as this height was achieved in all models. The selected trials (most similar G_0_) are marked (*) in Fig. 2.

Figure 4 shows muscle activities and joint kinematics for a 12 cm step-down for models with reduced muscle force (indicated with a * in Fig. 2). During level walking both MF-models showed increased muscle activities of all muscles compared to the default model (Fig. 4). When comparing level walking versus step-down with the SOL strategy, muscle activation of the SOL and GAS reduced, and activation in the VAS increased to a similar extent for all models (default model, 80_MF- and 60_MF-model; Fig. 4). The ankle and knee joint of the trailing leg were more flexed during the second half of the contact phase in all models (default model, 80_MF- and 60_MF-model; Fig. 4), resulting in similar alterations in knee and ankle kinematics. When the HAM strategy was used, the muscle activation of the HAM, VAS and SOL increased and the activation of the GAS decreased for all models during step-down compared to level walking (default model, 80_MF- and 60_MF-model; Fig. 4). As a result of the observed muscle adjustments, the ankle joint in all models was more plantarflexed at the end of the contact phase (Fig. 4).

**Fig. 5:**
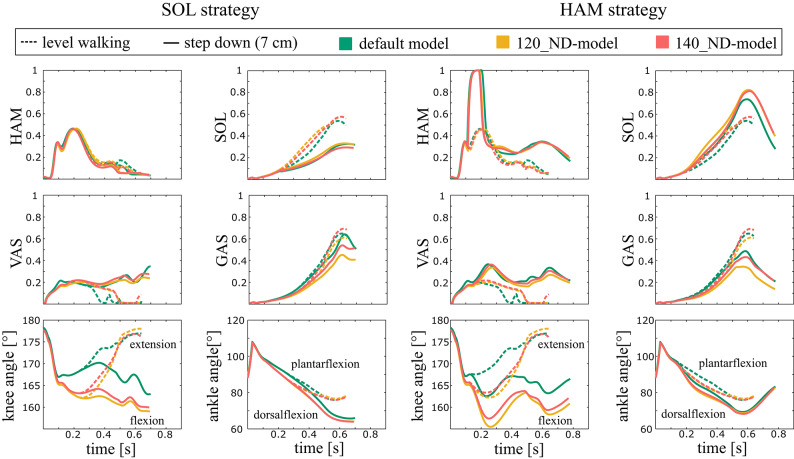
Muscle activities and kinematics during the preparatory contact phase (C_0_; 0 s ≙ heel strike) for the default and neural delay model variants. Different colors indicate the different levels of neural delay. Solid vs. dashed lines compare 7 cm step-down vs. level walking, respectively. Here, we show only the results for the 7 cm step-down, as this height was achieved in all models. The selected trials (most similar G_0_) are marked (*) in Fig. 3.

Figure 5 shows muscle activities and joint kinematics for a 7 cm stepdown, for models with increased neural delay. During level walking both ND-models showed no substantial changes in activation of the SOL, GAS, VAS and HAM (Fig. 5). Compared with the default model, both the 120_ND-model and the 140_ND-model showed more knee flexion during the contact phase. When comparing level walking versus step-down, the SOL strategy reduced muscle activation of the SOL and GAS and increased activation in the VAS for all models (default model, 120_ND- and 140_ND-model; Fig. 5). The ankle and knee joint of the trailing leg was more flexed during the second half of the contact phase in all models (default model, 120_ND- and 140_ND-model; Fig. 5). When the HAM strategy was applied, the muscle activation of the HAM, VAS and SOL increased and the activation of the GAS decreased for all models during step-down (default model, 120_ND- and 140_ND-model; Fig. 5). The ankle joint in all models was more plantarflexed at the end of the contact phase (Fig. 5).

## Discussion

Our predictive simulations show that anticipatory strategies involving the SOL and HAM are effective in improving maximal step-down in case of muscle weakness and increased neural delay. With increasing muscle weakness, both the maximum step-down height and the range of gain adjustments that led to successful step-downs decreased in the SOL strategy. In contrast, the HAM strategy’s performance was not affected by muscle weakness. For both strategies, increased neural delay led to a lower maximum step-down height, but the range of gain adjustments that led to successful step-downs remained similar. Our simulations indicate that the SOL strategy is favorable in most cases, but that in case of severe weakness, the HAM strategy could outperform the SOL strategy, as both the maximal step-down is higher and gain-range of robust solutions broader.

Unlike the HAM strategy, the gain-range of successful anticipatory SOL gain adjustments became smaller with increasing weakness, implying more precise selection of anticipatory muscle adaptations is required. When combined with other age-related sensorimotor changes, such as increased neural noise^24^ this reduced gain-range of robust solutions, combined with the lower maximal allowable step-down, could potentially increase the risk of falling. Moreover, step-down strategies rely on visual input^21,39–41^. Impairment of the visual system in older people^42^ may lead to inaccurate estimation of step-down height, making it more difficult to fine-tune the required anticipatory adjustments. When using the HAM strategy both the activation of the HAM and SOL muscles increases. This leads to a stiffer leg, as more muscles are activated (Fig. 5). Consequently, the step-down requires less precise muscle activation patterns across the muscles.

In older adults, with both muscle weakness and increased neural delay, higher levels of activation and co-contraction have been reported during step-down, indicating that they may indeed prefer the use of the HAM strategy. Reeves et al.^25^ and Karamanidis & Arampatzis^43^ showed that older people use the stronger knee flexors and extensors more than the plantar flexors when descending stairs. Furthermore, Buckley et al.^22^ observed higher co-contraction in the knee and ankle joints resulting in a more extended trailing leg during the descent of the first step of a staircase in older compared to younger individuals. These adaptations were also applied by the model when using the HAM strategy. The heightened HAM activity also increased the VAS activity in the reflex model (Fig. 5), which leads to a stronger co-contraction in the knee joint and a longer extended trailing leg during the preparatory contact (C_0_).

Despite this apparent preference in older adults for the more robust HAM strategy, our results also indicate overall advantages of the SOL strategy. In most cases (e.g., default model and with increased neural delay), the SOL strategy outperformed the HAM strategy, allowing for larger step-downs. Furthermore, increased co-contractions, as observed in the HAM strategy, are often associated with higher energy costs. The SOL strategy did not lead to increased co-contraction, which may indicate a more efficient gait in the case of repetitive step-downs, such as walking downhill or downstairs, or walking on uneven terrain. Hence, for older adults it may be favorable to keep making use of this SOL strategy for longer, for instance by training (calf) muscle strength to increase the gain-range of robust solutions. Further investigations are needed to confirm this.

Although experimental data align with the use of the HAM strategy, the extent to which this prediction can actually be transferred to older people still needs to be further examined. It would be relevant to study muscle activities and kinematics of older people during step-down walking experimentally in more detail, in relation to the levels of weakness and other age-related impairments. This data could be compared to those of younger people and serve to better validate the outcomes of our simulations. Furthermore, to gain further insight in the combined effect or age-related factors, the influence of the SOL and HAM strategies on the step-down robustness could be tested in a model variant with a combination of different age-related sensorimotor changes (e.g., muscle weakness, extended neural delay, neural noise). However, a pilot test demonstrated that the optimization problem could not be solved for a model variant with muscle weakness and extended neural delay, because the constraints (Δv, Δl, tc_diff_, S_diff_) of Eq. 1b for optimizing towards a steady state gait did not reach zero. Therefore, the target values and the weighting factors of the cost function would need to be modified for further investigation.

While a detailed sensitivity analysis was not part of this study, we systematically investigated the robustness of our anticipatory control strategies by varying key model parameters such as muscle strength and neural delays. In particular, we explored the effect of anticipatory gain modulation across a range of perturbation magnitudes to identify parameter ranges that yield robust gait solutions. The core model components and control framework have been validated in previous work^14^ where a parameter sensitivity analysis is reported.

Another topic of further study could be the potential adaptations in the leading leg. In the current implementation, the leading leg only reacts to the (delayed) ground contact and stabilizes gait with reflexes. However, studies^20,21,44,45^ show that the leading leg also plays an important role in compensating for stepping down. In the landing strategy of the leading leg, ankle initial contact angle and ankle range of motion are decisive parameters^44^. Additionally, in other experimental studies^22^ higher co-activities and preactivated muscles were also observed in the leading leg when older people descended stairs. It has already been shown that muscular adjustments in the leading leg can also increase the robustness of the reflex model^18^. To simulate pre-activation in the muscles of the leading leg, Haeufle et al.^18^ extended the model to include a feedforward control. In our current study, the full step height is compensated by anticipatory adjustments in the trailing leg and reflexes in the leading leg. However, further anticipatory adaptations in the leading leg may influence the level of required anticipatory control in the trailing leg. This potential interaction was not included in our simulations. A combination of adjustments in the leading and trailing legs could make the simulations more realistic and more robust.

## Conclusions

In conclusion, our predictive simulations suggest that in case of muscle weakness, the most effective anticipatory strategy for step-down shifts from a SOL to a HAM based strategy. In the simulations, the HAM based strategy required less precise muscle control, explaining the more robust behavior. Further experimental investigations are required to determine whether the HAM strategy is preferred by patients and whether training to maintain a SOL strategy may be beneficial.

## Data Availability

The datasets used and analyzed during the current study are available from the corresponding author on reasonable request.
